# Perspective: The Glycemic Index Falls Short as a Carbohydrate Food Quality Indicator to Improve Diet Quality

**DOI:** 10.3389/fnut.2022.896333

**Published:** 2022-04-20

**Authors:** Jill Nicholls

**Affiliations:** Food Context LLC, Indianapolis, IN, United States

**Keywords:** glycemic index, diet quality, dietary guidelines, dietary patterns, carbohydrate food quality, carbohydrates, nutrient-dense

## Abstract

This perspective examines the utility of the glycemic index (GI) as a carbohydrate quality indicator to improve Dietary Guidelines for Americans (DGA) adherence and diet quality. Achieving affordable, high-quality dietary patterns can address multiple nutrition and health priorities. Carbohydrate-containing foods make important energy, macronutrient, micronutrient, phytochemical, and bioactive contributions to dietary patterns, thus improving carbohydrate food quality may improve diet quality. Following DGA guidance helps meet nutrient needs, achieve good health, and reduce risk for diet-related non-communicable diseases in healthy people, yet adherence by Americans is low. A simple indicator that identifies high-quality carbohydrate foods and improves food choice may improve DGA adherence, but there is no consensus on a definition. The GI is a measure of the ability of the available carbohydrate in a food to increase blood glucose. The GI is well established in research literature and popular resources, and some have called for including the GI on food labels and in food-based dietary guidelines. The GI has increased understanding about physiological responses to carbohydrate-containing foods, yet its role in food-based dietary guidance and diet quality is unresolved. A one-dimensional indicator like the GI runs the risk of being interpreted to mean foods are “good” or “bad,” and it does not characterize the multiple contributions of carbohydrate-containing foods to diet quality, including nutrient density, a core concept in the DGA. New ways to define and communicate carbohydrate food quality shown to help improve adherence to high-quality dietary patterns such as described in the DGA would benefit public health.

## Introduction

DGA recommendations are meant to help meet nutrient needs, achieve good health, and reduce risk for diet-related non-communicable diseases (NCDs) in healthy people or people at risk for diet-related chronic diseases; they are not intended for disease treatment (1). Healthy eating patterns can also be a part of lifestyle modifications to address NCDs such as Type 2 Diabetes Mellitus (T2DM) and cardiovascular disease (CVD) (2, 3). DGA Healthy Dietary Patterns (HDPs) comprise “nutrient-dense forms of foods and beverages across all food groups, in recommended amounts, and within calorie limits” (1). In the U.S., diet quality is defined in terms of the Healthy Eating Index, i.e., how well a diet reflects DGA recommendations (4). Together, foods and beverages habitually consumed in various quantities, combinations, and proportions over time yield dietary patterns (5). Because dietary patterns include all foods and beverages, they can account for interactions between dietary components that influence metabolism and health (6). The quality of dietary patterns depends on their component foods and beverages, macro- and micronutrients, essential trace elements, plant-based phytochemicals and phytonutrients, and bioactive compounds (5). On average, Americans under consume nutrient-dense fruits, vegetables, whole grains, beans, and dairy foods and over consume nutrient-poor foods that contribute excess added sugars, saturated fat, and sodium (1). DGA adherence and thus diet quality of Americans remains low, and more than 60% of American adults have one or more diet-related NCD (1). There is interest, therefore, in tools to improve DGA adherence.

The food-based DGA HDPs include carbohydrate-containing foods in all major food groups, and carbohydrates contribute ~50% of calorie intake in the U.S. DGA carbohydrate-related guidance includes meeting recommendations for under consumed nutrient-dense foods (e.g., fruits, vegetables, whole grains, milk, and legumes); limiting sugar-sweetened beverages; increasing foods high in dietary fiber; and meeting Acceptable Macronutrient Dietary Ranges for carbohydrate, fat, and protein (1). Carbohydrate-containing foods also contribute flavor and texture attributes to foods and beverages. The diversity of carbohydrate-containing foods and beverages adds complexity to defining carbohydrate food quality in a way that will complement DGA guidance and improve adherence.

## Carbohydrate Food Quality

Carbohydrate-containing staple foods are important parts of HDPs, yet sometimes staples are also identified as foods to reduce or avoid in research literature and popular resources (7–11). Consumer confusion may result when foods that are affordable, accessible, and acceptable are not the foods recommended as part of HDPs. Of the 50% of dietary energy from carbohydrate-containing foods in the U.S., the main contributors are refined grains (16% of carbohydrates) and added sugars (14% of carbohydrates) (12). These general food categories may not be helpful for describing quality. Whole and refined (or enriched) staple grain foods can be part of HDPs as foods like tortillas, bread, or cereal, while more indulgent foods made from refined grains such as cookies and cakes are foods to limit (13).

Common indicators of carbohydrate food quality include dietary fiber, added sugars, nutrient density, and GI. The GI is a measure of the ability of the available carbohydrate in a food to increase blood glucose. It emerged in the 1980s to help people with Type 1 Diabetes modify their food choices to manage blood glucose and insulin (14), and it expanded the understanding of the physiological effects of carbohydrates beyond simple and complex designations. Low-GI foods (≤55) are digested and absorbed slowly, and high-GI foods (≥70) are digested and absorbed more quickly.

The GI is based on a food's available carbohydrate content and can be influenced by food processing and preparation methods, physical and chemical characteristics such as acidity or starch type and content (15), and the presence of protein, fat, and fiber (15–20). Foods such as pasta, dairy, legumes, and some fruits have GI values in the low-GI category (14, 20). Staple high-carbohydrate foods such as bread, grains, and potatoes span from low- to high-GI values (20). The GI range for “rice products,” for example, is 19–116, with a mean GI for white rice of 73 and for brown rice of 65. The range for boiled potatoes is 38–103, with a mean GI of 73 (20). Differences for staples may be due to variety, cooking or processing methods, or storage temperature (20).

The GI has been associated with reduced risk for T2DM and CHD (21, 22), and combination indicators for carbohydrate quality have been linked with positive health outcomes. Carbohydrate-to-fiber ratios are associated with lower risk for T2DM (23) and CVD (24). The Carbohydrate Quality Index (CQI) accounts for dietary fiber, GI, whole grains-to-total grains ratio, and solid-to-liquid carbohydrates ratio (25). Higher CQI is associated with lower risk for obesity (26), CVD (27), and CVD risk factors (28). Comparisons between CQI and carbohydrate-to-fiber ratios associated with waist circumference change found carbohydrate-to-fiber and carbohydrate-to-cereal fiber ratios were better predictors than CQI (29). One carbohydrate indicator may not fit all situations.

Low-GI dietary patterns are not necessarily low-carbohydrate, though low-GI and low-carbohydrate concepts can overlap. Low-carbohydrate diets are not a core topic of this article, however, because they are not part of DGA recommendations. The 2020–2025 Dietary Guidelines Advisory Committee reviewed evidence about dietary patterns based on macronutrient distribution and found limited evidence for such diets reducing risk for CVD, and insufficient evidence to establish a relationship between these diets and T2DM; growth, size, body composition and risk of overweight or obesity; sarcopenia; and mortality (5).

## Dietary Guidelines for Americans, GI, and Health Outcomes

Adherence to the DGA is associated with reduced risk for NCDs. The DGA HDPs reflect findings from extensive evidence reviews on dietary patterns and multiple health outcomes (5). Strong evidence links DGA HDPs with reduced risk for all-cause mortality and CVD, and moderate evidence links HDPs with improved growth, body composition and body weight; reduced risk for T2DM; and bone health (5). The 2015 and 2020–2025 DGA do not recommend the GI (5, 30), and the 2010 DGA found strong evidence that the GI and glycemic load (GL), the product of a food GI and the quantity of available carbohydrate in a serving of that food, “are not associated with body weight; thus, it is not necessary to consider these measures when selecting carbohydrate foods and beverages for weight management” (31).

Prolonged exposure to elevated blood glucose is a risk factor for T2DM (32) and glycemic control is an important factor in metabolic health, but the relevance of the GI to guide food choice for HDPs—the role of the DGA—is unresolved (33–35). A series of systematic reviews and meta-analyses of prospective cohort studies using the carbohydrate quality indicators dietary fiber, whole grains, and the GI assessed the relationships between these indicators and several NCDs (36). Observational evidence indicated higher dietary fiber intakes were associated with a 15–30% decrease in all-cause and CVD mortality and incidence of CHD, stroke, and T2DM. The certainty of evidence was graded as moderate for dietary fiber, low to moderate for whole grains, and low to very low for dietary GI and GL (36). This evidence supports DGA guidance to consume foods high in dietary fiber and whole grains. The GI is not included by any national food agency in major food-based dietary guidelines (FBDGs) (37), though some countries allow GI labeling (38).

## Dietary Guidelines for Americans, GI, and Nutrient Adequacy

The DGA recommendations help people meet nutrient needs by including nutrient-dense versions of foods from all food groups in HDPs that contribute specific groups of macro- and micronutrients to influence health (1). Together, foods are part of HDPs based on multiple attributes; no single food, macronutrient, or micronutrient can provide the attributes needed for good health.

Low-GI foods are not necessarily nutrient-dense, so adherence to the DGA is a more reliable way to ensure nutrient adequacy than consuming a low-GI diet. Over-reliance on GI values may lead to choices inconsistent with DGA recommendations. Some low-GI foods are energy-dense due to fat and/or sugar content and should be consumed in moderation (e.g., ice cream, cookies, and candy bars), while some high-GI foods are nutrient-dense and part of HDPs (e.g., carrots, potatoes, and grains).

Nutrient adequacy has been associated with carbohydrate food quality metrics including the GI (39, 40), carbohydrate ratios (41–43), and CQI scores (25, 44). These relationships may be present because nutrients and other dietary components are correlated in foods (45). In a cohort consuming the Mediterranean dietary pattern, for example, the lowest probability of nutrient inadequacy was found in the highest quintile of both the *a priori* MeDiet score and the CQI (25), likely due to adherence to the Mediterranean dietary pattern.

## More Clincial Trials About Diet Quality and GI are Needed

Many studies have examined chronic disease risk in terms of low- and high-GI diets, low- and high-GL diets, or low- and high-adherence to dietary patterns such as Mediterranean, Dietary Approaches to Stop Hypertension (DASH), or DGA. Few trials, however, have modified GI or GL of high-quality dietary patterns. Two well-known randomized controlled trials studied CVD biomarkers (46) and weight loss (47) in this context and found high-quality dietary patterns had unexpected impacts on outcomes (46, 47).

In the OmniCarb trial, the high-quality DASH diet was modified to test the effect of low- and high-GI foods in low- and high-carbohydrate dietary patterns on CVD risk factors in 163 overweight adults (46). Participants consumed each diet for 5 weeks. Contrary to their hypothesis, researchers concluded that a DASH-type diet containing low-GI foods compared with high-GI foods “does not improve CVD risk factors and may in fact reduce insulin sensitivity” (46).

The Diet Intervention Examining the Factors Interacting with Treatment Success (DIETFITS) study was a large, 12-month study of “healthy” diets that were either low-fat (51% carbohydrates; high-GL) or low-carbohydrate (27% carbohydrate; low-GL), compared for their effect on weight loss in 609 adults (47). All participants received guidance to choose high-quality, nutrient-dense foods including more vegetables; fewer added sugars, refined flours, and *trans* fats; and more minimally processed, nutrient-dense whole foods. The DIETFITS study found no difference in average weight loss between the diet groups, an outcome that differed from previous studies comparing low-fat and low-carbohydrate diets. The researchers indicated that the high quality of both diets, not simply their macronutrient differences, was a key contributor to findings (47).

More trials that shed light on the interactions between diet quality, nutrient-dense carbohydrate foods, and incidence of NCDs can aid understanding of diet quality impacts. Some evidence indicates that consuming vegan, vegetarian, DASH and DGA dietary patterns may improve glycemic control in people with T2DM compared to conventional diets for T2DM management (48). In prospective cohort studies, higher adherence to high-quality dietary patterns has been linked to less weight gain over time (49, 50), reduced risk for T2DM (51) and stroke mortality (52), and lower mortality from all causes and CVD (53–55).

## GI Values May Not Predict Meal Glycemic Responses

Because the GI model is based on individual foods analyzed under standard conditions, its application may be limited for characterizing health-promoting dietary patterns including foods consumed in real-world conditions. In studies that determined the GI of staple foods like rice, potatoes, or pasta alone and as part of mixed meals containing typical amounts of protein (chicken or egg), fiber (vegetables), and oil or sauce, the measured GIs of mixed meals were lower than predicted based on food GIs by 20–50% (56–59). Different approaches to calculating mixed meal GIs have been proposed (60). Calculated meal GIs may not agree with measured meal GIs without accounting for protein, fat, and available carbohydrate (60), a practice unrealistic for general consumer use. The GI is considered a property of foods that does not change when eaten with other foods, but food GI values may not be sufficient information to predict post-meal GRs. Eating balanced meals or dietary patterns provides an avenue to modify GRs not apparent from GI values.

Another way to account for meal and diet GRs is GL, developed to evaluate diets in epidemiological studies (61, 62). The GL is the product of the food GI and the quantity of available carbohydrate in a serving of that food. Accurate GL population estimates depend on accurate food intake information and correct food GI values. Given the broad ranges in GIs of staple carbohydrate foods, and food frequency questionnaire inaccuracies, combining GL and food intake may exacerbate inaccuracies in prospective cohort study data about GL and health. Even assuming researchers calculate population GLs using accurate food intake and GI data, the approach would not account for mixed meal GRs described above.

## The GI is a Property of Food

GIs are physiological responses measured in human subjects; thus, they are determined using a fundamentally different measurement approach than direct analysis of food components like nutrients or fiber. Its reliability has been questioned since its introduction, including critiques about methodology, the many things that can influence measurements, and the relationship between food GIs and post-meal GR (60). One unresolved topic is the role of within- and between-individual variation during GI determination (33–35, 60, 63). GI methodology was developed to minimize within-person variation during measurement. The International Organization for Standardization method, for example, requires repeated testing in at least 10 subjects under highly-controlled conditions to minimize within-individual variation (64).

Another perspective about GRs has emerged from studies about personalized nutrition that collected detailed information about glycemic and other metabolic responses to foods and meals in hundreds of subjects (35, 65). The study by Zeevi et al. measured postprandial GR to meals in 800 subjects (65), and the study by Berry et al. measured postprandial glucose, triglyceride, and insulin responses to meals in 1,002 individuals (35), in a laboratory and at home. Both groups of researchers found metabolic responses to the same meals were highly variable *between* individuals, but they were very similar *within* individuals and predictable based on person-specific characteristics (emphasis added) (35, 65). Though this interpretation of GRs was challenged (66), the consistent findings of predictable individual GRs led authors to question the utility of identifying dietary ingredients as universally “good” or “bad” (65) or recommending universal nutrition recommendations (35) based on population averages.

## Meal Context is a New Determinant of Postprandial Metabolism

Berry et al. examined sources of variance in individual postprandial metabolic responses. The variance attributable to genetic heritability explained 0% of the variance for triglyceride, and 48% for glucose, thus about half of the variance in GRs is not modifiable (35). Meal macronutrient composition (carbohydrates, sugar, fat, protein, and fiber) explained about 17% of variance, a similar percentage to “meal context,” which included non-food, meal-related factors such as meal timing, exercise, sleep, and circadian rhythm (35). Meal composition and context were “core determinants” of individual postprandial metabolism.

Personalized nutrition is not a reality for most people but studying metabolic responses to meals in real-world conditions may provide important insights about the interplay between dietary patterns and metabolic health beyond GI values or macronutrient content. The National Institutes of Health Precision Nutrition Program aims to develop algorithms to explain the “interactions between diet, genes, proteins, microbiome, metabolism, and other individual contextual factors” in response to different diets (67). The program made its first research awards in January 2022.

## Discussion

The DGA and other FBDGs have been evolving and continue to adapt to new evidence and priorities. In 1990, one of the DGA's seven recommendations was to eat no more than 30% of calories from fat and 10% from saturated fat, and another was to eat more breads, cereals, pasta, and rice (68). Thirty years later, the 2020–2025 DGA are flexible food-based guidance organized by life stage, are informed by research on dietary patterns associated with positive health outcomes and can accommodate macronutrient ranges rather than require specific targets (1). Consuming DGA HDPs is associated with multiple positive health outcomes, but adherence is low. Because carbohydrates contribute about half of dietary calories in the U.S., tools to improve carbohydrate food quality may help improve overall diet quality of Americans.

FBDGs are also prioritizing access to affordable, high-quality dietary patterns for those who are food insecure (69) and to healthy diets from sustainable food systems (70). In populations consuming traditional diets with a high proportion of low-cost carbohydrate staple foods, improving diet quality may mean increasing intakes of nutrient-dense foods without disparaging affordable, accessible, culturally acceptable staples. In other populations, it may mean trading nutrient-poor choices high in added sugars, saturated fat, and sodium for more nutrient-dense choices while reducing energy intake. The search for sustainable dietary approaches to food and nutrition security may increase interest in traditional or innovative plant-based and carbohydrate-containing foods eaten in new contexts. These priorities have implications for defining carbohydrate food quality and communicating its role in diet quality and health.

Quality is difficult to define. Diet quality includes aspects of adequacy, moderation, and balance (71), but there is not a consensus about what constitutes high-quality carbohydrate foods and beverages. Single quality indicators fall short due to the diversity of carbohydrate foods. Saying high quality carbohydrate foods should all be high-fiber, sugar-free, or low-GI will not adequately characterize carbohydrate foods needed to build a healthy diet. Food quality indicators used within a dietary framework like the DGA can better guide food choice. The GI does not address nutrient density, it does not translate well to HDPs, and its singular focus on one dimension of carbohydrate-containing foods may divert public attention away from dietary patterns-based approaches to improving health. In addition, among common measures of carbohydrate food quality used in regulatory frameworks, the effects of carbohydrate-containing foods on postprandial glycemia are “the most contentious” (72).

Carbohydrate food quality is multi-dimensional. Hypotheses about carbohydrates in health and disease are changing in ways that support new, possibly composite, carbohydrate food quality indicators (35, 36, 47, 73, 74). Current efforts to improve diet quality would benefit from new approaches to define and communicate carbohydrate food quality that complement food-based guidance affordably, holistically, and sustainably.

## Data Availability Statement

The original contributions presented in the perspective are included in the article; further inquiries can be directed to the corresponding author.

## Author Contributions

The author confirms being the sole contributor of this work and has approved it for publication.

## Funding

This perspective was supported by funds from the Alliance for Potato Research and Education. The funder was not involved in the interpretation of data, the writing of this article or the decision to submit it for publication.

## Conflict of Interest

JN is the owner of Food Context, LLC, where she provides food and nutrition consulting services to the food and beverage industry.

## Publisher's Note

All claims expressed in this article are solely those of the authors and do not necessarily represent those of their affiliated organizations, or those of the publisher, the editors and the reviewers. Any product that may be evaluated in this article, or claim that may be made by its manufacturer, is not guaranteed or endorsed by the publisher.
